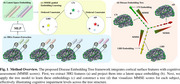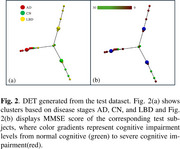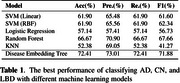# Representing the Cognitive Impairment Continuum of Alzheimer's Disease and Lewy Body Dementia with a Novel Finer‐Scale Cortical Representation via Disease Embedding Tree

**DOI:** 10.1002/alz70856_102179

**Published:** 2025-12-24

**Authors:** Tong Chen, Minheng Chen, Yan Zhuang, JIng Zhang, Lu Zhang, Tianming Liu, Andrew M Blamire, John T O'Brien, Li Su, Dajiang Zhu

**Affiliations:** ^1^ The University of Texas at Arlington, Arlington, TX, USA; ^2^ University of Texas at Arlington, Arlington, TX, USA; ^3^ Indiana University Indianapolis, Indianapolis, IN, USA; ^4^ University of Georgia, Athens, GA, USA; ^5^ Newcastle University, Newcastle Magnetic Resonance Centre, Newcastle upon Tyne, United Kingdom; ^6^ University of Cambridge, Cambridge, United Kingdom; ^7^ University of Sheffield, Sheffield, United Kingdom

## Abstract

**Background:**

Alzheimer's Disease (AD) and Lewy Body Dementia (LBD) often exhibit overlapping neuropathological features and symptoms, posing significant challenges for differential diagnosis. While many studies focus on leveraging machine learning with neuroimaging data for early dementia diagnosis, investigating the progression and interactions between AD and LBD offers an opportunity to uncover valuable insights into their shared features and hidden connections.

**Method:**

We propose the Disease Embedding Tree (DET) framework to model continuous relationships among AD, Cognitively Normal (CN), and LBD subjects on T1‐weighted structural MRI data from 106 subjects (36 AD: 15 females, 21 males; 78.25 ± 5.76 years; 35 CN: 15 females, 20 males; 76.74 ± 5.15 years; 35 LBD: 8 females, 27 males; 78.37 ± 6.94 years).

For each subject, we reconstructed cortical surfaces and adopted a novel cortical folding pattern representation, Gyral Network, to identify the potential cortical hubs, known as 3 hinge gyri (3HGs). Cortical features, including cortical thickness, curvature, sulcal depth, fractal dimension, and local gyrification index, were extracted from 3HGs and used to train the DET model.

The DET model projects the extracted features of each subject to a high‐dimensional embedding space. The order constraint conditions the inter‐group relationship while the Mini‐Mental State Examination (MMSE) scores are incorporated to model the inter‐subject continuous relationship in embedding space, shown in Figure 1. Additionally, our model supports the classification task based on the proximity of the subjects in the embedding space, providing both continuous relationship and diagnostic capability.

**Result:**

The DET framework has achieved superior performance on the classification task across all the evaluation metrics compared to the traditional machine learning based methods, as demonstrated in Table 1. Figure 2 shows that the CN, AD patients, LBD patients are projected to the DET based on the learned representations in the embedding space.

**Conclusion:**

The DET framework effectively models the continuous relationships among CN, AD, and LBD subjects and outperforms the traditional models in the classification task. By leveraging cortical features of the cortical hubs and the MMSE‐based constraints, it provides valuable insights into disease progression.